# Thymoquinone attenuates diabetes-induced hepatic damage in rat via regulation of oxidative/nitrosative stress, apoptosis, and inflammatory cascade with molecular docking approach

**DOI:** 10.1038/s41598-024-62780-y

**Published:** 2024-06-06

**Authors:** Mona H. Hafez, Samar M. Ez Elarab, Hossam G. Tohamy, Ali H. El-Far

**Affiliations:** 1 Department of Physiology, Faculty of Veterinary Medicine, Alexandria University, Alexandria, 22758 Egypt; 2 Department of Histology and Cytology, Faculty of Veterinary Medicine, Alexandria University, Alexandria, 22758 Egypt; 3 Department of Pathology, Faculty of Veterinary Medicine, Alexandria University, Alexandria, 22758 Egypt; 4 Department of Biochemistry, Faculty of Veterinary Medicine, Damanhour University, Damanhour, 22511 Egypt

**Keywords:** Molecular docking, Oxidative stress, Pro-inflammatory cytokines, Streptozotocin, Thymoquinone, Biochemistry, Medical research

## Abstract

Diabetes mellitus (DM) is a complex metabolic condition that causes organ dysfunction. The current experiment sought to determine the effect of thymoquinone (TQ) on hyperglycemia, hyperlipidemia, oxidative/nitrosative stress, inflammation, and apoptosis in diabetic rats prompted by streptozotocin (STZ) (55 mg/kg body weight i/p). The animals were allocated into control, TQ (50 mg/kg B.W. orally administered for 4 succeeding weeks), Diabetic, and Diabetic + TQ groups. This study confirmed that TQ preserves the levels of insulin, fasting blood glucose, HOMA *β*-cell indices, HbA1c %, body weight, and lipid profile substantially relative to the DC group. Furthermore, hepatic antioxidant (CAT, GSH, and T-SOD) values were reduced. Conversely, the enzymatic activity of liver functions (AST, ALT, ALP, cytochrome P450, and hepatic glucose-6-phosphatase), lipid peroxidation (MDA), pro-inflammatory cytokines (IL-1*β*, TNF-*α*, and IL-6), nitric oxide (NO) and inflammatory marker (CRP) enhanced with STZ administration, which is substantially restored after TQ treatment. Relative to the diabetic rats, TQ reestablished the hepatic architectural changes and collagen fibers. Additionally, TQ downregulated the intensity of the immunohistochemical staining of pro-apoptotic marker (caspase-3), p53, and tumor necrosis factor-alpha (TNF-*α*) proteins in hepatic tissues. Furthermore, TQ displayed abilities to interact and inhibit the binding site of caspase-3, interleukin-6 receptor, interleukin-1 receptor type 1, TNF receptor superfamily member 1A, and TNF receptor superfamily member 1B in rats following the molecular docking modeling. All these data re-establish the liver functions, antioxidant enzymes, anti-inflammatory markers, and anti-apoptotic proteins impacts of TQ in STZ-induced DM rats. Founded on these outcomes, the experiment proposes that TQ is a novel natural supplement with various clinical applications, including managing DM, which in turn is recommended to play a pivotal role in preventing the progression of diabetes mellitus.

## Introduction

Natural ingredients have traditionally occupied a role in drug development, and they also served as the foundation for the earliest medications. In folk medicine, they were used to treat a variety of diseases and illnesses. Several plants have been discovered to offer anti-diabetic characteristics^1,2^. Furthermore, various phytoconstituents with anti-diabetic properties have been identified in plants in recent years. Thymoquinone (TQ; 2-isopropyl-5-methyl-1, 4-benzoquinone) is a bioactive phytochemical ingredient of *Nigella sativa* seeds' volatile oil. TQ is a commonly safe constituent, particularly when given orally to laboratory animals^3^. TQ has been shown to have different pharmacological actions, including anti-inflammatory, immunomodulatory, anti-diabetic, antioxidant, anticancer, and anti-aging properties^4–15^. Previous research has shown that TQ has a substantial antioxidant effect against a variety of free radical-producing chemicals, including diabetes-induced testicular atrophy^10^ and cardiac^11^ alterations, cisplatin-induced hepato-toxicity, doxorubicin-induced cardiotoxicity, and cadmium-induced reproductive dysfunctions^16,17^. Furthermore, TQ was also shown to protect against diabetic nephropathy and membrane-induced lipid peroxidation^2,18^.

Islets of Langerhans organelles are found in the pancreas and are responsible for synthesizing insulin, glucagon, somatostatin, and pancreatic polypeptide in response to stimulation^3^. Diabetes mellitus (DM) is a chronic, serious, and complicated metabolic syndrome, and the primary goal of islet research is to develop a cure and improve DM care^19^. The diabetes population is growing fast at an alarming rate and is expected to reach over 600 million by 2035^2^. Diabetes triggers long-term failure, dysfunction, and damage of several important organs, including the liver^1^. One of the primary organs impacted by DM is the hepatic tissue function, and resistance to insulin follows in both liver and peripheral tissues, resulting in unrestrained hepatic glucose levels and impaired uptake of peripheral glucose^20^. DM complications have long been recognized, including oxidative stress caused by persistent and chronic hyperglycemia, which increases free radical production, stress of endoplasmic reticulum, and inflammation, causing cell injury via apoptosis in many tissues, including the hepatic tissue^2,8^. Additionally, reactive oxygen species (ROS): hydroxyl radicals, nitric oxide (NO), and superoxide radicals are harmful chemicals that contribute to cellular death. ROS causes cellular harm by damaging the cells' biological components, such as peptides, DNA, and lipids, and hence death of the cell^16^. Diabetic liver damage is thought to be caused primarily by inflammation and disruptions in systemic and hepatic fat metabolism. Furthermore, systemic hyperglycemia changes carbohydrate metabolism in the liver, exacerbating the disruption in peripheral glucose uptake^21^. Hyperglycemia and reduced glucose tolerance are linked to a variety of hepatic, metabolic, vascular, nephropathic, and neuropathic dysfunctions. The major indicators for detecting DM metabolic problems are hyperglycemia and lipid profile anomalies^22^. Hypertriglyceridemia, low levels of high-density lipoprotein-cholesterol (HDL-C), and high values of low-density lipoprotein-cholesterol (LDL-C) are all related to DM and contribute considerably to the development of atherosclerosis^1^. When investigating the efficacy of anti-diabetic medications, antioxidant properties, efficacy in treating dyslipidemia and hyperglycemia, and safety are fundamental^16^. To limit the risks of diabetic complications, effective diabetes care necessitates continuous control of blood sugar levels. Thus, therapeutic and natural antioxidants are one of the diabetic treatment techniques^3,23^. For the best of our research, only a few experiments were undertaken to investigate the impact of TQ on DM-induced liver damage. So, the current work aimed to evaluate the impact of TQ on glycemic control, oxidative stress, hepatic functions biomarkers, lipid profile, molecular docking, histological and immunohistochemical staining of caspase-3, p53, and tumor necrosis factor-alpha (TNF-*α*) proteins in hepatic tissues of streptozotocin (STZ)-triggered diabetic rats.

## Materials and methods

### Ethics statement

All experimental protocols were approved by Animal Research. Animal Care Review Committee of the Faculty of Veterinary Medicine, Alexandria University, Alexandria, Egypt, accepted the current study following the rules for the maintenance and use of laboratory animals (Committee permit number: 2022/013/167). All methods were carried out following animal research guidelines and regulations. All experiments were conducted in accordance with the ARRIVE guidelines^24^.

### Chemicals and reagents

STZ (Product#: 572,201) and TQ (Product#: 274,666; ≥ 98%) were bought from Sigma–Aldrich (St. Louis, MO, USA). Hemoglucotest® glucose strips were acquired from Roche Diagnostics (Montreal, Canada). Hemoglobin A1c (HbA1c) %, glucose, malondialdehyde (MDA) analyze kit, reduced glutathione (GSH), total serum nitrate/nitrite kit, CAT kits, total superoxide dismutase (T-SOD), and serum aspartate aminotransferase (AST), alanine aminotransferase (ALT), and alkaline phosphatase (ALP) concentration kits were obtained from Biodiagnostic (Cat. Tahrir, Cairo, Egypt). Rat's insulin enzyme-linked immunosorbent assay (ELISA) analysis kit (Cat#; 80-INSRTH-E01, E10 American Laboratory Products Co., USA). Proinflammatory cytokines (IL-6, IL-1*β*, and TNF-*α*) ELISA kits (Anogen, Mississauga, Ontario, Canada). Triacylglycerol (TAG), total cholesterol (TC), and LDL-C and HDL-C triggering chemical kits were obtained from United Diagnostics (Cairo, Egypt). Entire chemicals consumed were of analytical grade and were utilized as obtained without any additional purification.

### Animals

In this experiment, 32 Sprague–Dawley male rats, 180–200 g of average weight, were employed. Rats were housed in plastic cages (each group was housed in a cage of 50 × 50 cm and a height of 30 cm with a wood shaving bedding, applied to a depth of 2 cm in each cage) and fed a regular laboratory ration containing 0.5% NaCl, 22% protein, and 4–6% dietary fat (Damanhour Feed Co, Behera, Egypt) with free access to water. Throughout the study, rats were housed at a normal room temperature of 22–25°C, with humidity and a light cycle. Animals were obtained from Alexandria University's Medical Research Institute and adapted to laboratory settings for 2 weeks.

### Induction of diabetes

The rats were distributed into 4 equal groups (*n* = 8 for every group). In groups 3 and 4, DM was produced with a single intraperitoneal infusion of STZ (55 mg/kg body weight) freshly liquefied in 5 mM citrate buffer, pH 4.5^20^. Control rats (groups 1 and 2) were infused with an identical amount (1 mL) of buffer solution only. To dismiss the STZ-triggered hypoglycemia, animals were permitted to receive an overnight 5% sugar solution. Following three days, fasting blood glucose levels were immediately determined using Hemoglucotest® glucose strips (Roche Diagnostics, Montreal, Canada). Three days after STZ administration, animals with more than 200 mg/dL of blood glucose were rendered diabetic^25^. Treatment was introduced on the 3rd day after STZ injection, which was considered experiment 1st day. Consequently, all animals were sustained for 4 weeks (the entire experimental period) on ad libitum water and food with inspection of average body weight, food, and water ingestion, and fasting blood glucose levels prior to the start of TQ administration^8^.

### Experimental design

Rats were allocated to 4 groups at random, each with eight rats:

i. Rats in group 1 (control group) were administrated with 1 mL of distilled water / 100 g of BW/day through gastric gavage.
ii. Rats in group 2 (TQ group) were administered with TQ, firstly dissolved by the addition of dimethyl sulfoxide, then by adding normal saline (for a final dimethyl sulfoxide value of less than 0.5%). Subsequent TQ solution was given at 50 mg/kg of BW one time per day by oral gavage for up to four weeks^8^. The dosage was accustomed weekly following any alteration in body weight to sustain a parallel dose per kg BW of rats over the whole experimental period for every group.
iii. Rats in group 3 (Diabetic) were rendered diabetic as previously described and were administered 1 mL of distilled water / 100 g of BW/day by gastric gavage.
iv. Rats in group 4 (Diabetic + TQ) were rendered diabetic and administrated with TQ as defined with group 2.

### Body weight measurements

The body weights of all animals were measured at the start of each week, and findings were analyzed accordingly.

### Oral glucose tolerance test (OGTT)

After ending the experiment, animals were deprived overnight and then given glucose solution (2 g of glucose/ 5ml distilled water/kg Bwt of rats) intragastrically. Samples of the blood were drawn from the tail vein at 0, 30, 60, 90, and 120 min. Glucose levels were evaluated immediately in the blood samples by Hemoglucotest® glucose strips^20,24,26^.

### Blood and tissue assembly and preparation

Twenty-four hours following the last treatment administration, with ketamine/xylazine anesthesia (7.5 and 1.0 mg/kg via intra-peritoneal infusion)^23^, 2 samples of the blood were collected from the heart on sodium fluoride at room temperature. One sample was utilized to calculate HbA1c%, and the other was centrifuged at 1,800 × g for 15 min to separate plasma for insulin, glucose, liver function tests, lipid profile, and inflammatory marker analysis.

Following blood collection, and while the animals were still anesthetized, euthanasia was performed via decapitation, and dissected, each liver excised and thoroughly estimated. A part of the hepatic tissue of every rat was washed with deionized water and physiological saline solution (NaCl 0.9%) to remove RBCs and platelets; tissues were blotted with blotting paper and perfused with 50 mM sodium phosphate saline buffer (100 mM Na_2_HPO_4_/NaH_2_PO_4_, pH 7.4) in an ice-cold medium with 0.1 mM EDTA. Then, tissues were shredded in 10 mL of ice-cold buffer/g tissue and centrifuged for 30 min at 10,000 × g. The supernatant was transferred to Eppendorf tubes and stored at -80°C until oxidative/nitrosative and anti-oxidative enzyme activities were determined. Another section of liver tissue was removed and promptly preserved in 10% buffered formaldehyde for histological and immunohistochemical analysis.

#### Blood biochemical measurements

Plasma glucose concentrations^27^ (Cat#: BD-30234) and HbA1c %^28^ (Cat#: BD-10453) were spectrophotometrically evaluated through provided analysis kits (Bio-diagnostic Co., Cairo, Egypt). A High Range Rat’s Insulin ELISA kit (Cat#: 102-INSRTH-E01, E10 American Laboratory Products Co., USA) was consumed to analyze the plasma insulin values.

#### Homeostasis model assessment of β-cell function (HOMA-β-cell) index

For HOMA *β*-cell index determination, plasma insulin, and fasting glucose levels were utilized according to Matthews's formula^29^:

$${\text{HOMA }}\beta - {\text{cell Index}} = \left( {20 \times \frac{{{\text{Fasting plasma insulin }}\left( {{\upmu }\frac{{\text{U}}}{{{\text{ml}}}}} \right)}}{{{\text{Fasting plasma glucose }}\left( {\frac{{{\text{mmol}}}}{{{\text{ml}}}}} \right)}}} \right){-}{3}.{5}$$

Insulin sensitivity indices: fasting glucose/insulin ratio and insulin-1 were calculated from plasma insulin and fasting glucose concentrations^30^.

#### Liver function tests and lipid profile measurements

ALT (Cat#: DD0440), AST (Cat#: DD0453), and ALP (Cat#: DD0363) content were estimated using commercially available kits (Diamond Diagnostics Co., Cairo, Egypt) as per the manufacturer's directions. The activity of plasma's cytochrome p450 (CYP450) (Cat#: BD34701) was determined through ELISA kits. Markers of lipid profile, including TAG, TC, HDL-C, and LDL-C, were analyzed by kits (Bio-Diagnostics Co., Cairo, Egypt), and the data were interpreted consequently. The atherogenic index in plasma (AIP) was determined through the formula = log (TAG/HDL-C)^22^.

#### Analysis of proinflammatory cytokines and inflammatory marker

Interleukin-6 (IL-6), TNF-*α*, and interleukin-1*β* (IL-1*β*), as proinflammatory cytokines and inflammatory marker C reactive protein (CRP) were estimated in the plasma following manufacturer's procedures of obtained ELISA kits (Cat#: 102-ALPD-A201, E510 and A218 respectively, Millipore, CA, USA). To confirm all readings, an ELISA Plate Reader (Bio-Rad, Hercules, CA, USA) was consumed.

#### Hepatic lipid peroxidation, antioxidants, and G6Pase

Homogenates of hepatic tissues were utilized to calculate malondialdehyde (MDA) (Cat#: BD0523) levels^31^. NO was measured indirectly by determining nitrite synthesis in hepatic extract using the Griess diazotization reaction^32^, reduced glutathione (GSH) (Cat#: BD0385)^33^, T-SOD (Cat#: BD0342) and catalase (CAT) (Cat#: BD0422) enzyme activities^34^ were determined using provided diagnostic examine kits (Bio-diagnostic Co., Cairo, Egypt). The activity of glucose-6-phosphatase (G6Pase) in the liver was colorimetrically valued following Barman^35^. The content of protein in hepatic tissues was measured^36^.

### Histopathological examination and semi‑quantitative lesion scoring

Hepatic tissue samples were obtained from 8 rats per group and promptly maintained in buffered formalin 10% for a minimum of 24 h. Hepatic samples were washed, dehydrated with serial alcohol dilutions, cleaned in xylene, and inserted in paraffin at 60°C. To assess the extent of collagen, paraffin slices of five microns thickness were produced and stained with hematoxylin and eosin (H and E) and Masson's trichrome stain, then inspected beneath a light microscope. The extent of hepatic tissue damage was evaluated using a semiquantitative scoring assay, in which five random fields were examined from each section. The severity of lesions was scored and graded according to the percentage of affected tissue, as follows: none (-) = 0%, representing no involvement of the examined field; mild ( +) = 5–25% of the examined field; moderate (+ +) = 25–50% of the examined field; and severe (+ + +) ≥ 50–100% of the examined field^37^.

### Immunohistochemical evaluation

Following the manufacturer's directions, a standard horseradish peroxidase-immunohistochemical approach employing rabbit anti-rat p53, caspase-3, and TNF-*α* was smeared to positively charged slides of paraffin Sects^38^. Five-micron slices of hepatic tissue were dewaxed, rehydrated, and incubated with 3% hydrogen peroxide to inhibit endogenous peroxidase activity. The slides were antigen-retrieved by putting them in a microwave oven for 10 min in a 10 mM sodium citrate buffer (pH 6.0). Then, endogenous peroxidase activity was blocked with 3% H_2_O_2_ for 10 min. Nonspecific proteins were inhibited by 2% bovine serum albumin. The slices were rinsed three times in Dako Tris-buffered saline before being incubated overnight at 4°C with a primary rabbit polyclonal anti-p53 antibody (1:100) (PA5-32,045; Thermo-Fisher Scientific, WA, USA), rabbit polyclonal anti-caspase-3 antibody (1:100) (Code# ab4051; Abcam, Cambridge, UK) and rabbit polyclonal anti- TNF-*α* antibody (1:100) (PA5-41,057; Thermo-Fisher Scientific, WA, USA). The tissue slices were rinsed in Tris-buffered saline and then incubated with the streptavidin–horseradish peroxidase reagent and a biotinylated secondary antibody for 30 min at 37°C. Slides were then treated with a 3, 3' diaminobenzidine substrate chromogen solution, counterstained with Mayer's hematoxylin, and photographed. Images of 10 different fields, at a magnification of × 400, were analyzed using ImageJ software to estimate the area (%) of caspase3, P-53 and TNF-α positive brown immunostained cells^37^.

### Molecular docking

#### Ligand preparation

The three-dimensional (3D) structure of TQ was retrieved from the PubChem (https://pubchem.ncbi.nlm.nih.gov/) database in SDF format and opened in MOE 2015.10^39^ software for energy minimization and docking with target proteins.

#### Protein preparation

Caspases-3, interleukin-1 receptor type 1 (IL1R1), TNF receptor superfamily member 1A (TNFRSF1A), and TNF receptor superfamily member 1B (TNFRSF1B) 3D structures from rats were acquired from UniProt database (https://www.uniprot.org/). While the 3D structure of the interleukin-6 receptor (IL-6R) was produced by the Robetta server^40^. Target proteins were prepared for docking using MOE software along with target protein energy minimization.

#### Molecular docking analysis and visualization

Target proteins were docked with TQ using MOE software and the protein–ligand interactions were visualized by the same software.

### Statistical analysis

GraphPad Prism v.9 (https://www.graphpad.com/) (GraphPad, San Diego, CA, USA) analyzed the data by a one-way ANOVA with Tukey's post hoc multiple range testing. Two-way ANOVA analyzed body weight and OGTT data with Tukey's post hoc multiple range testing. *P* < 0.05 was required for all significance declarations.

## Results

### Body weights

**Figure 1:**
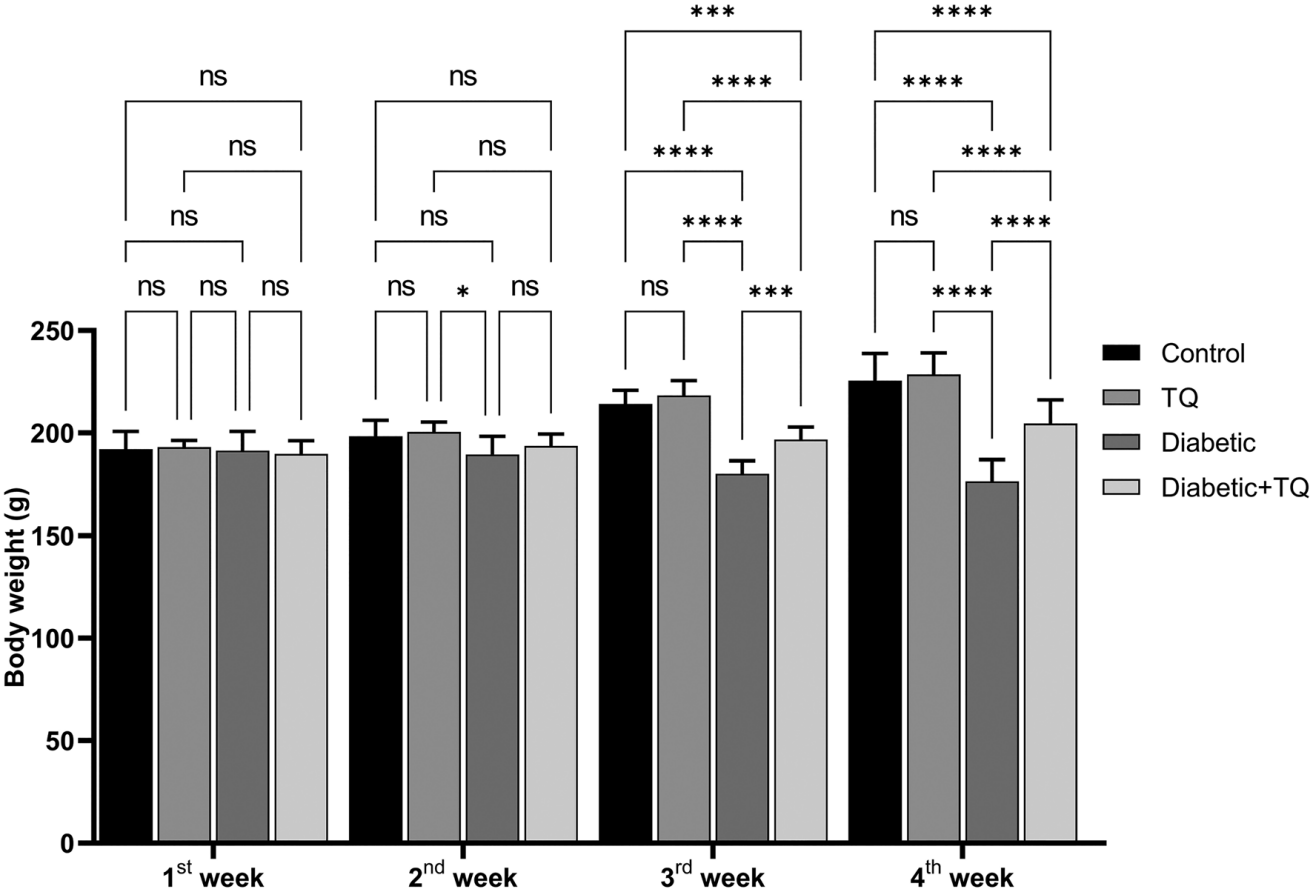
Assessment of body weight. Data were analyzed with a two-way ANOVA followed by Tukey’s multiple comparison test. Data are expressed as the mean ± SD. *n* = 8. ns = nonsignificant, ^***^P ˂ 0.001, and ^****^P ˂ 0.0001.

Weights of the body of the STZ-induced diabetic animals decreased markedly (*P* < 0.001) than those of the control non-diabetic group in the 3rd and 4th weeks of the study. However, TQ-treated diabetic rats improved the weights of the body (*P* < 0.001) relative to the diabetic non-treated animals in the 3rd and 4th weeks of the study. A non-significant variation was documented between the weights of control non-diabetic and TQ-treated rats (Fig. 1).

### Oral glucose tolerance test

**Figure 2:**
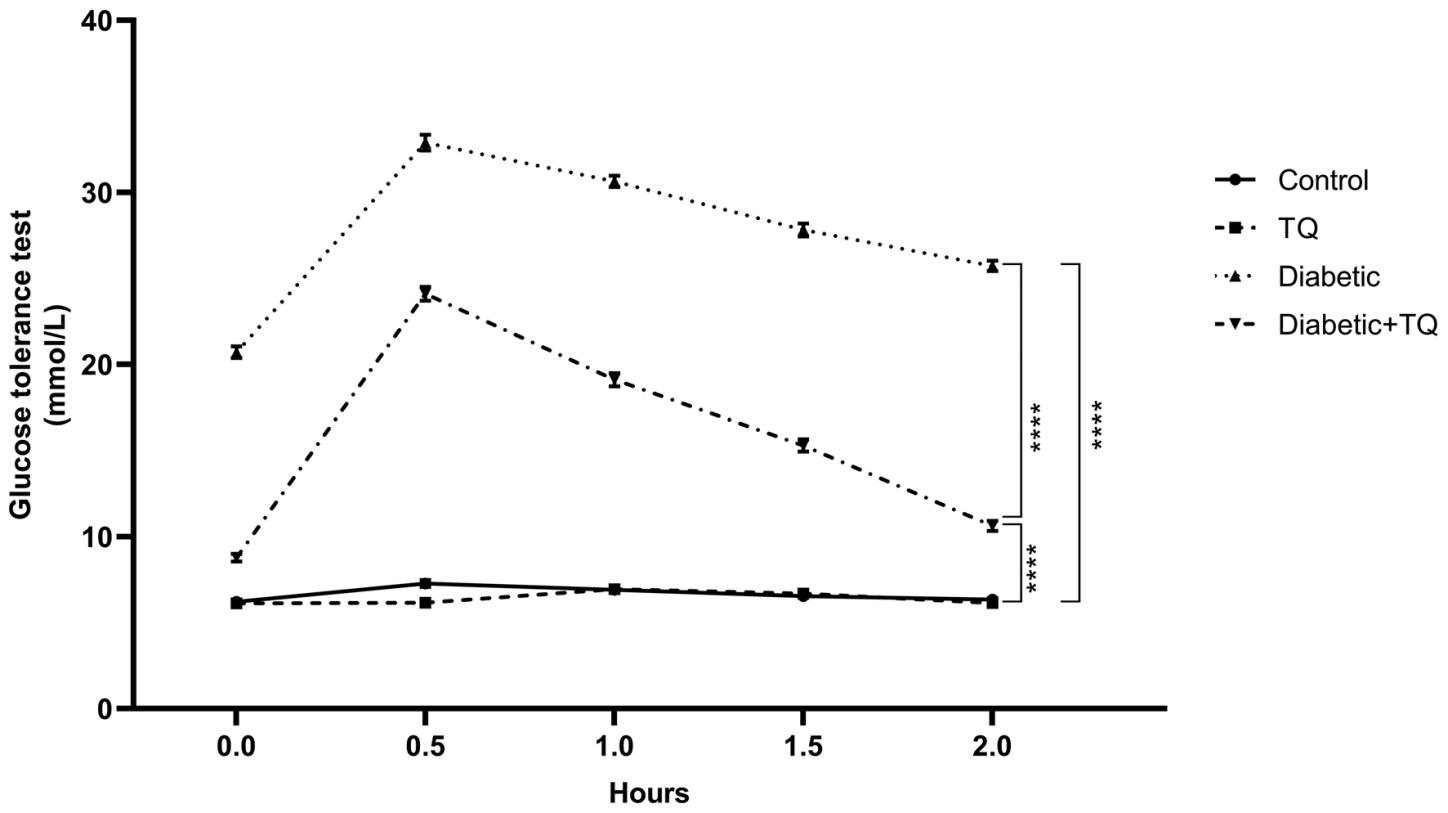
Assessment of glucose tolerance test. Data were analyzed with a two-way ANOVA followed by Tukey’s multiple comparison test. Data are expressed as the mean ± SD. *n* = 8. ^****^P ˂ 0.0001.

Differences in blood glucose levels of all groups throughout various time courses (0–120 min) are displayed in Fig. 2. The maximum value of glucose was identified after 30 min of receiving glucose (2 g/kg B.W.) and then reduced to main levels within 2 h in all experimental animals except STZ-diabetic control, which displayed an extreme glucose intolerance (*P* < 0.001) throughout the entire time course (0–120 min). Nonetheless, diabetic animals treated with TQ had substantially (*P* < 0.001) lower glucose levels than the normal control group. During the various time courses of the oral glucose tolerance test, there was no significant variation in blood glucose concentrations of the control non-diabetic and TQ-treated rats.

### Blood glycemic parameters and β-cell function indices

**Figure 3:**
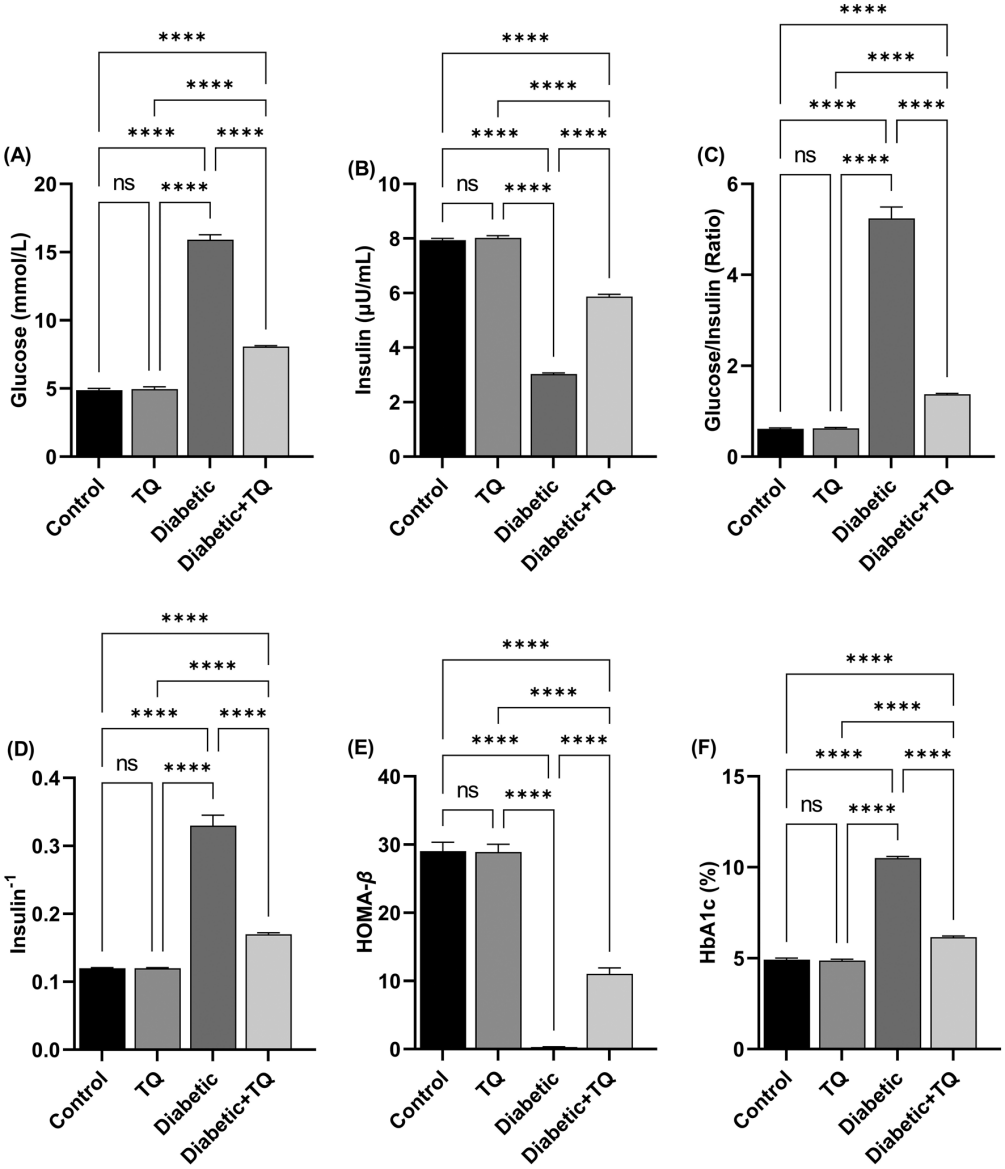
Assessment of blood glycemic parameters and *β*-cell function indices. **(A)** Glucose. **(B)** Insulin. **(C)** Glucose/insulin ratio. **(D)** Insulin^−1^. **(E)** HOMA-*β*. **(F)** hemoglobin A1c (HbA1c) %. Data were analyzed with a one-way ANOVA followed by Tukey’s multiple comparison test. Data are expressed as the mean ± SD. *n* = 8. ns = nonsignificant and ^****^P ˂ 0.0001.

As revealed in Fig. 3, compared to non-diabetic control animals, there were statistically marked (*P* < 0.001) increases in fasting plasma glucose (Fig. 3A), glucose/insulin ratio (Fig. 3C), HbA1c% (Fig. 3F) and insulin^−1^ (Fig. 3D) in the diabetic group. Acquired data also displayed that there was a substantial reduction in HOMA*β*-cell index (Fig. 3E) and fasting insulin (Fig. 3B) value in STZ-diabetic rats as relative to non-diabetic control animals. Those parameters improved significantly in diabetic TQ-treated rats relative to diabetic control rats, with fasting plasma glucose, insulin^−1^, HbA1c%, and glucose/insulin ratio significantly (*P* < 0.001) lowered in diabetic animals treated with TQ relative to the diabetic group. While the HOMA-cell index and fasting insulin were markedly (*P* < 0.001) higher in the Diabetic + TQ treated animals relative to the Diabetic group.

### Hepatic function tests and lipid profile

**Figure 4:**
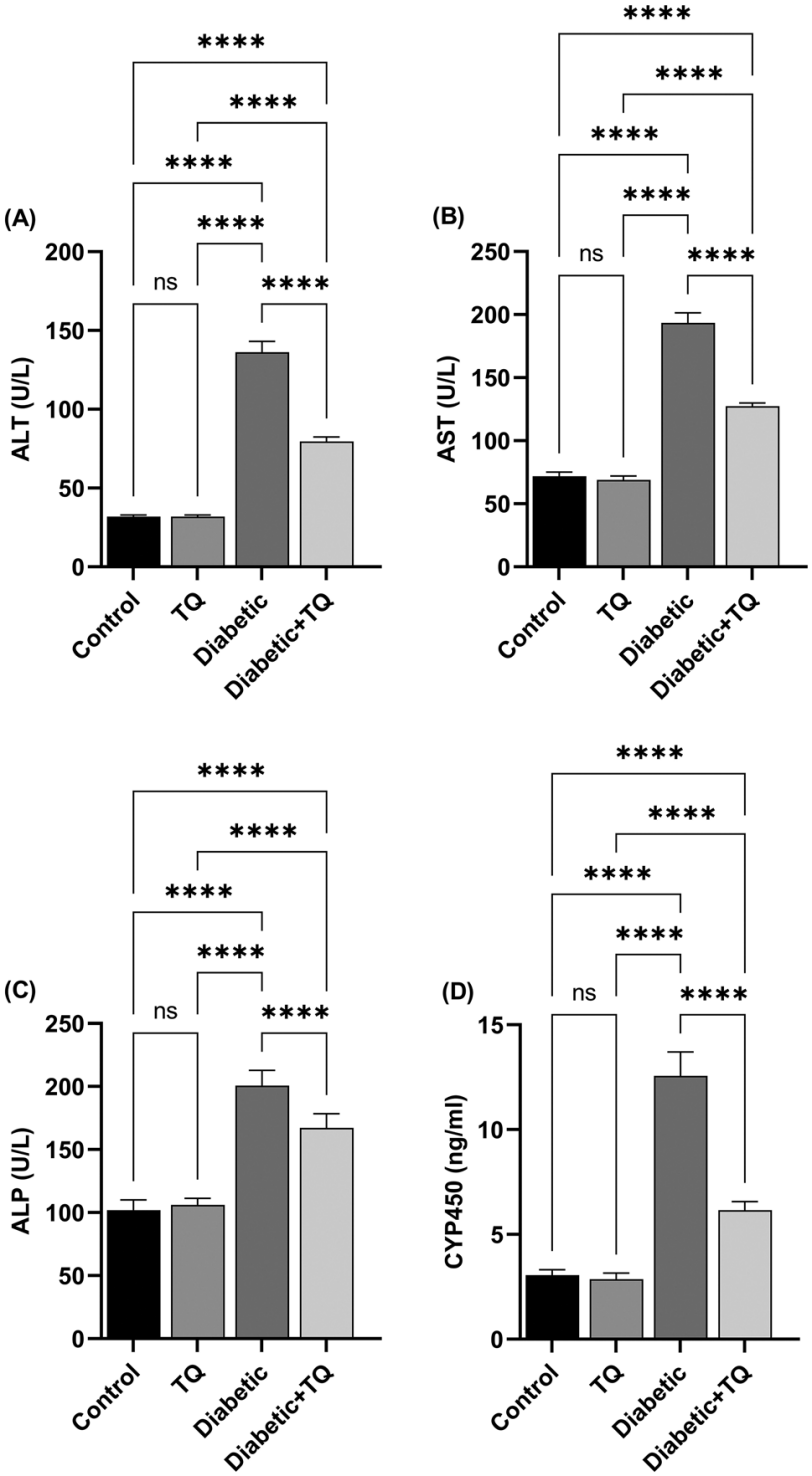
Assessment of blood hepatic function and hepatic glucose-6-phosphatase (G6Pase) activity*.* **(A)** Alanine aminotransferase (ALT). **(B)** Aspartate aminotransferase (AST). **(C)** Alkaline phosphatase (ALP). **(D)** Cytochrome p450 (CYP450). Data were analyzed with a one-way ANOVA followed by Tukey’s multiple comparison test. Data are expressed as the mean ± SD. *n* = 8. ns = nonsignificant, ^*^P ˂ 0.05, and ^****^P ˂ 0.0001.

Our findings revealed that STZ-induced diabetes induced a marked (*P* < 0.001) enhancement in plasma ALT (Fig. 4A), AST (Fig. 4B), and ALP (Fig. 4C) values, which indicates a hepatic tissue injury and destruction of the hepatocyte cell membrane. Moreover, it also enhanced plasma CYP450 (Fig. 4D). TQ therapy of diabetic rats reduced these effects substantially (*P* < 0.001) reducing hepatic function tests and CYP450 activity relative to diabetic control rats. The hepatic function tests and CYP450 activity were not markedly variant between the TQ-treated group and the control non-diabetic one.

**Figure 5:**
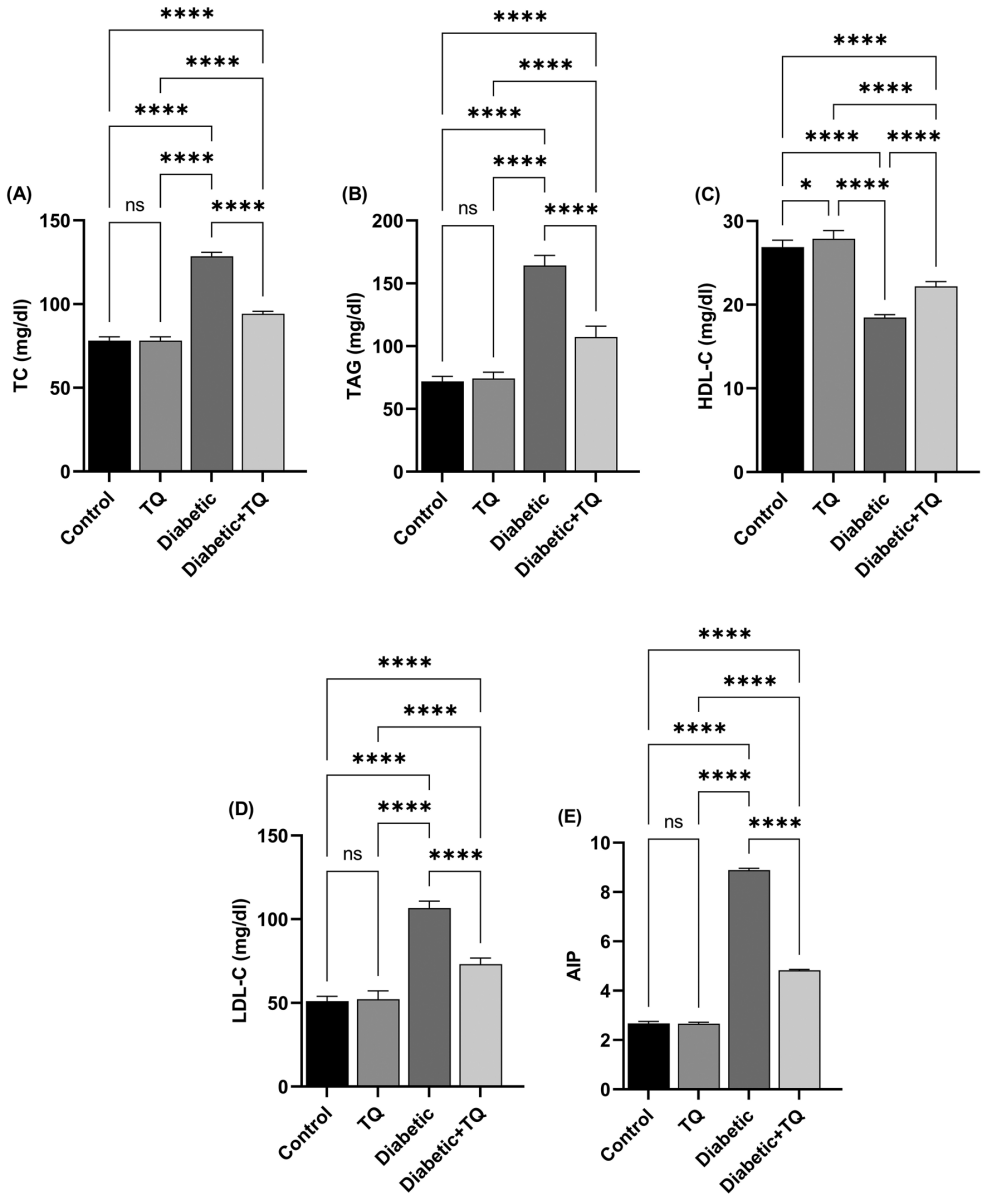
Assessment of lipid profile and atherogenic index (AIP)*.* **(A)** Total cholesterol (TC). **(B)** Triacylglycerol (TAG). **(C)** High-density lipoprotein-cholesterol (HDL-C). **(D)** Low-density lipoprotein-cholesterol (LDL-C). **(E)** Atherogenic index (AIP). Data were analyzed with a one-way ANOVA followed by Tukey’s multiple comparison test. Data are expressed as the mean ± SD. *n* = 8. ns = nonsignificant, ^*^P ˂ 0.05, and ^****^P ˂ 0.0001.

Figure 5 displays the lipid profile and atherogenic index in normal and experimental rats in each group. STZ-induced diabetes triggered a marked (*P* < 0.001) enhancement in plasma TC (Fig. 5A), TAG (Fig. 5B), LDL-C (Fig. 5D), and atherogenic index (Fig. 5E) values with a marked reduction in HDL-C (Fig. 5C). Treating diabetic animals with TQ led to a marked (*P* < 0.001) down-regulation in plasma TC, LDL-C, TAG, and atherogenic index values relative to the diabetic group. TQ enhanced the marked reduction in HDL-C of diabetic animals. A non-significant difference was recorded between the control non-diabetic and TQ-treated rats considering the lipid profile and atherogenic index except for the HDL-C which was reduced (*P* < 0.05) in the TQ group chart compared to the control one.

### Pro-inflammatory cytokines and inflammatory marker

**Figure 6:**
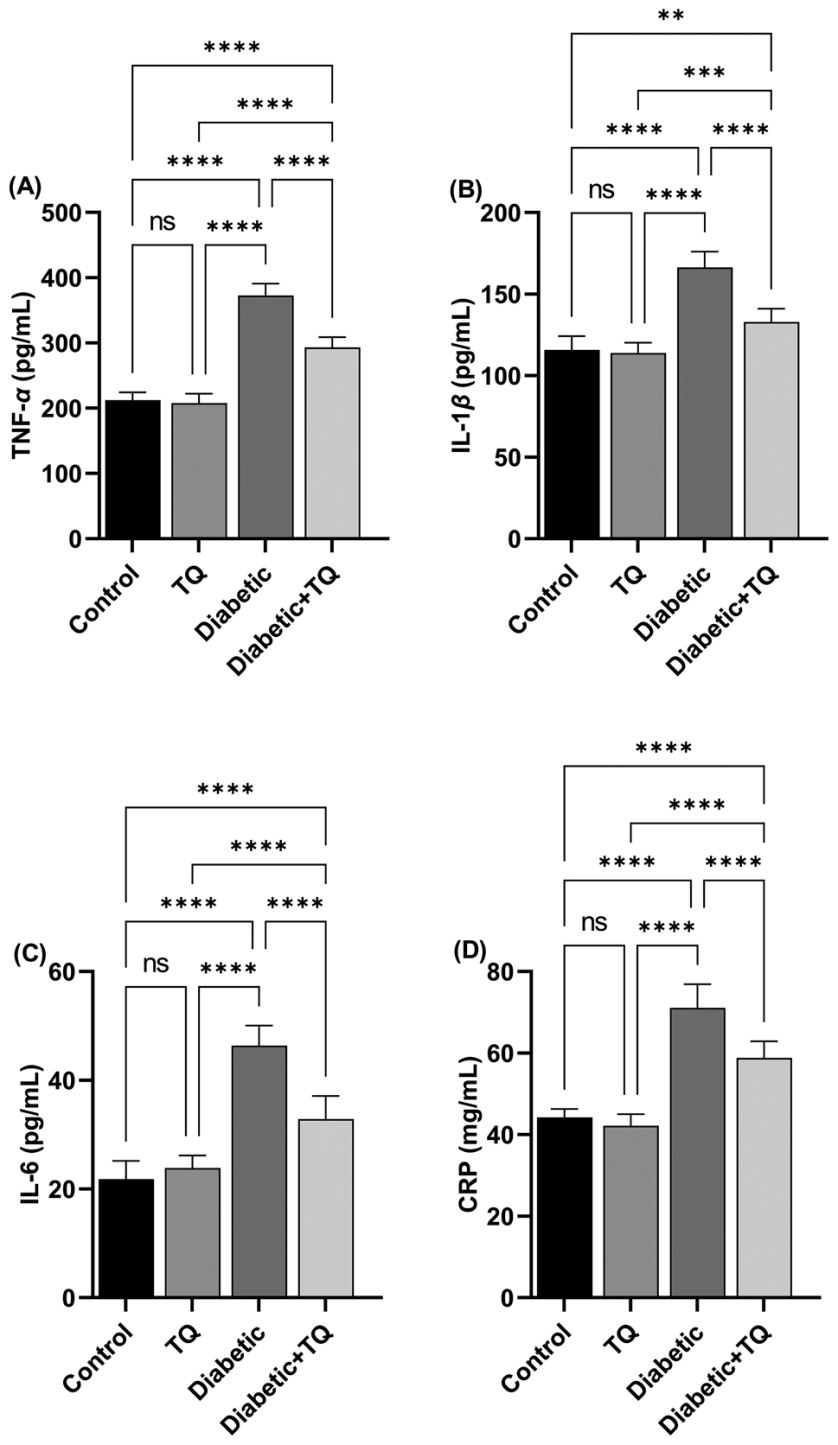
Assessment of plasma proinflammatory cytokines and inflammatory markers*.* **(A)** Tumor necrosis factor-alpha (TNF-*α*). **(B)** Interleukin-1*β* (IL-1*β*). **(C)** Interleukin-6 (IL-6). **(D)** C reactive protein (CRP). Data were analyzed with a one-way ANOVA followed by Tukey’s multiple comparison test. Data are expressed as the mean ± SD. *n* = 8. ns = nonsignificant, ^**^P ˂ 0.01, ^***^P ˂ 0.001, and ^****^P ˂ 0.0001.

STZ-induced diabetic control rats displayed a marked (*P* < 0.001) enhancement in plasma concentrations of TNF-*α* (Fig. 6A), IL-1*β* (Fig. 6B), IL-6 (Fig. 6C), and CRP (Fig. 6D) relative to the normal control group, which indicate the higher grade of the inflammatory cascade in the liver through diabetes. Nevertheless, TQ administration reduced markedly (*P* < 0.001) degree of enhanced proinflammatory, and inflammatory markers relative to diabetic control rats. A non-significant variation was documented in proinflammatory, and inflammatory biomarkers between the control non-diabetic and TQ-treated rats.

### Oxidant-antioxidant status and G6Pase

**Figure 7:**
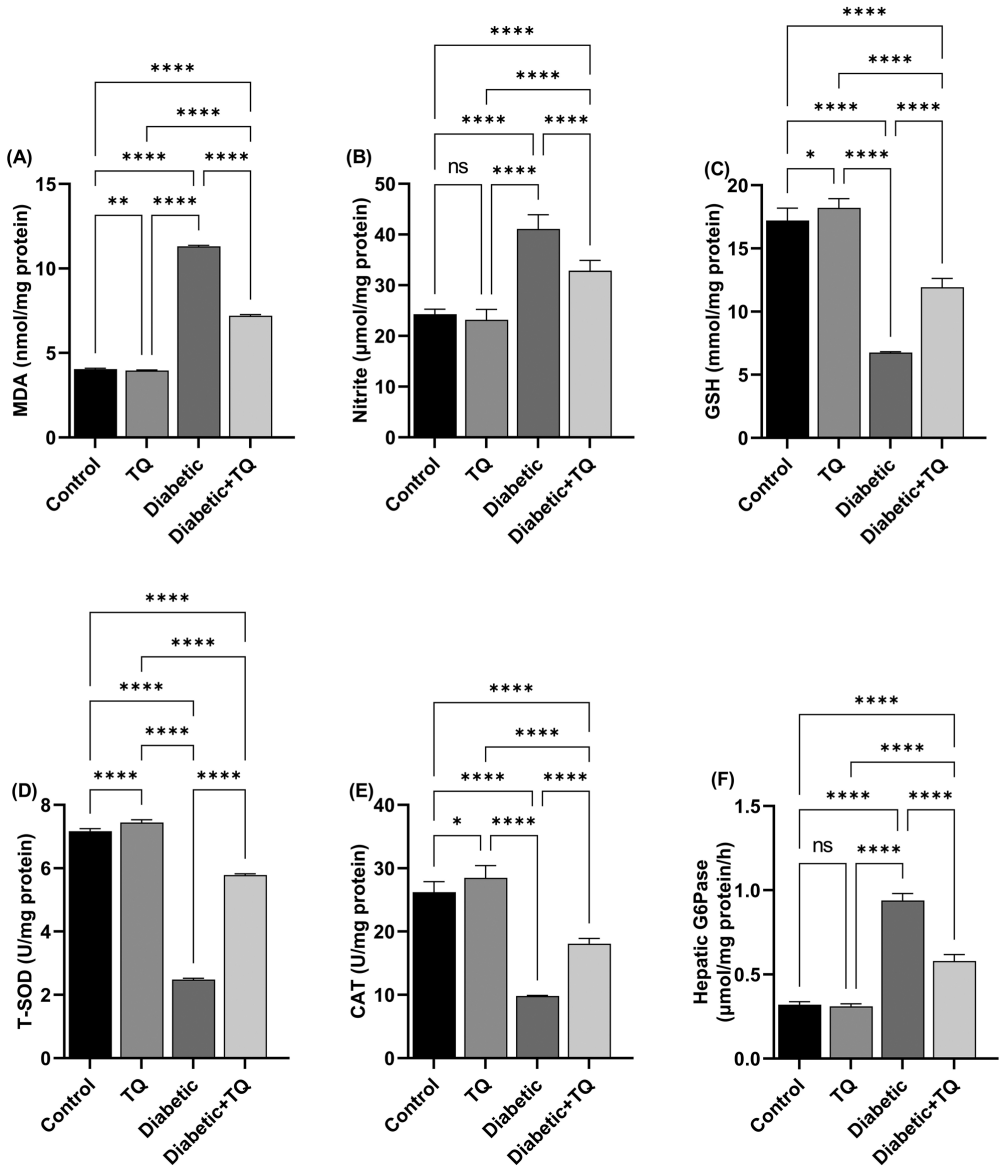
Assessment of hepatic lipid peroxidation, antioxidants, and G6Pase*.* **(A)** Malondialdehyde (MDA). **(B)** Nitric oxide (NO). **(C)** Reduced glutathione (GSH). **(D)** Total superoxide dismutase (T-SOD). **(E)** Catalase (CAT). **(F)** Glucose-6-phosphatase (G6Pase). Data were analyzed with a one-way ANOVA followed by Tukey’s multiple comparison test. Data are expressed as the mean ± SD. ns = nonsignificant, ^*^P ˂ 0.05, ^**^P ˂ 0.01, ^***^P ˂ 0.001, and ^****^P ˂ 0.0001.

The diabetic group showed a substantial (*P* < 0.001) enhancement in hepatic MDA (lipid peroxidation marker) (Fig. 7A), nitrite (Fig. 7B) values, and the activity of hepatic G6Pase (Fig. 7F); which is linked with a marked reduction in GSH (Fig. 7C) value and T-SOD (Fig. 7D) and CAT (Fig. 7E) activities relative to the normal control group. On the contrary, TQ treatment of diabetic rats markedly (*P* < 0.001) reduced the degree of increased LPO, nitrosative markers, and G6Pase hepatic activity relative to the diabetic control animals. Additionally, relative to diabetic rats, the antioxidant markers were mostly recovered in the Diabetic + TQ animals. TQ alone administrated rats revealed a marked (*P* < 0.01) reduction in intensity of MDA and nitrite concentrations relative to non-diabetic control animals. Furthermore, the antioxidant enzymatic activities in the TQ-treated rats were considerably (*P* < 0.01) enhanced than in normal control animals. Regarding the hepatic G6Pase activity, there was a non-marked variation between the TQ-treated group and the control non-diabetic one.

### Histopathological findings

**Figure 8:**
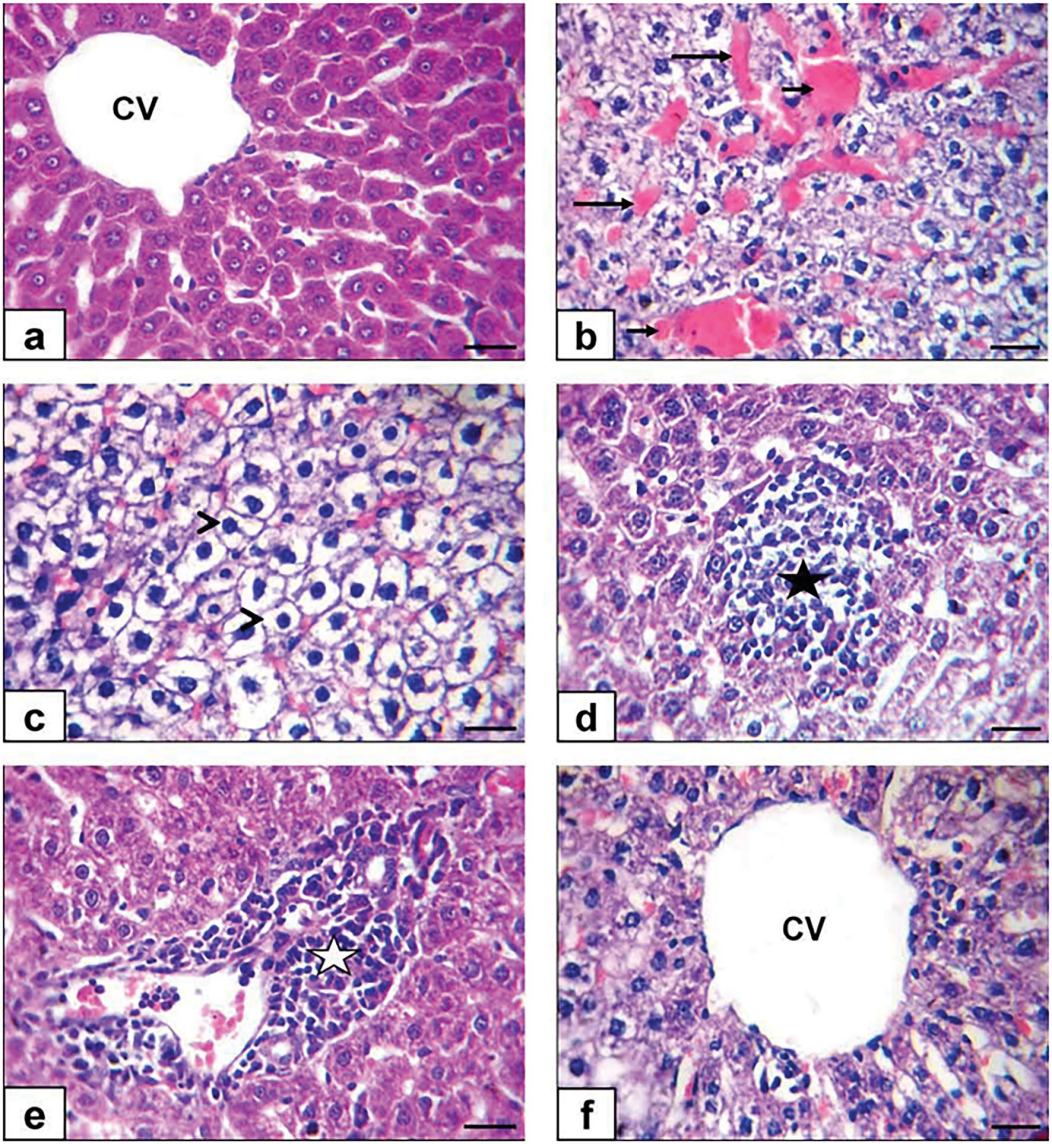
Photomicrograph of a rat liver section stained by H and E × 400. (**a**) Control rats showing normal histology with normal central vein (CV) and hepatocytes (**b**, **c**, **d**, **e**) diabetic rats showing congestion of hepatic sinusoid (long black arrows) and central vein (short black arrows), diffuse hydropic degeneration of hepatocytes (arrowheads) beside hepatocellular necrosis with mononuclear cell infiltrations (black star) and intense mononuclear cell infiltrations in portal area (white star) (**f**) TQ + diabetic treated rats showing normal hepatic histoarchitecture with normal central vein (CV). (Scale bar = 50 μm).

No histological variations were observed between non-diabetic control and TQ-administrated rats in the liver. So, they were deliberated as control. Control and TQ-treated rats' hepatic tissue displayed a normal histological structure, with undamaged hepatic architecture in hepatic lobules, portal areas, and hepatic vasculature (Fig. 8A). meanwhile, hepatocytes of the diabetic group exhibited diffuse congestion of hepatic vasculature (hepatic sinusoids and central vein) (Table 1 and Fig. 8B), diffuse hepatic hydropic degeneration where the cells were swollen, and the cytoplasm being replaced by clear fluids (Table 1 and Fig. 8C) alongside hepatocellular necrosis with mononuclear cell infiltration (Table 1 and Fig. 8D) and intense mononuclear cell infiltrations in the portal area (Table 1 and Fig. 8E). Simultaneously, hepatic tissue sections of the Diabetic + TQ group displayed nearly normal histoarchitecture (Table 1 and Fig. 8F).

**Table 1:** Incidence and severity of hepatic histopathological lesions in the experimental groups.

Group ⁄ lesion	Incidence and severity of histopathological lesions															
	control				TQ				Diabetic				Diabetic + TQ			
	−	+	+ +	+ + +	−	+	+ +	+ + +	−	+	+ +	+ + +	−	+	+ +	+ + +
Congestion of hepatic vasculature	6	2	0	0	7	1	0	0	0	1	2	5	5	3	0	0
Hepatic hydropic degeneration	7	1	0	0	7	1	0	0	0	1	3	4	6	2	0	0
hepatocellular necrosis	8	0	0	0	8	0	0	0	0	2	3	3	7	1	0	0
Mononuclear cell infiltrations in the portal area	8	0	0	0	8	0	0	0	1	1	3	3	7	1	0	0

Number of animals with lesions per total examined (8 rats per group). TQ Thymoquinone.

Severity of lesions was graded by estimating the percentage area affected in the entire section. Lesion scoring: (−) absence of the lesion = 0%, ( +) mild = 5–25%, (+ +) moderate = 26–50% and (+ + +) severe ≥ 50% of the examined tissue sections.

**Figure 9:**
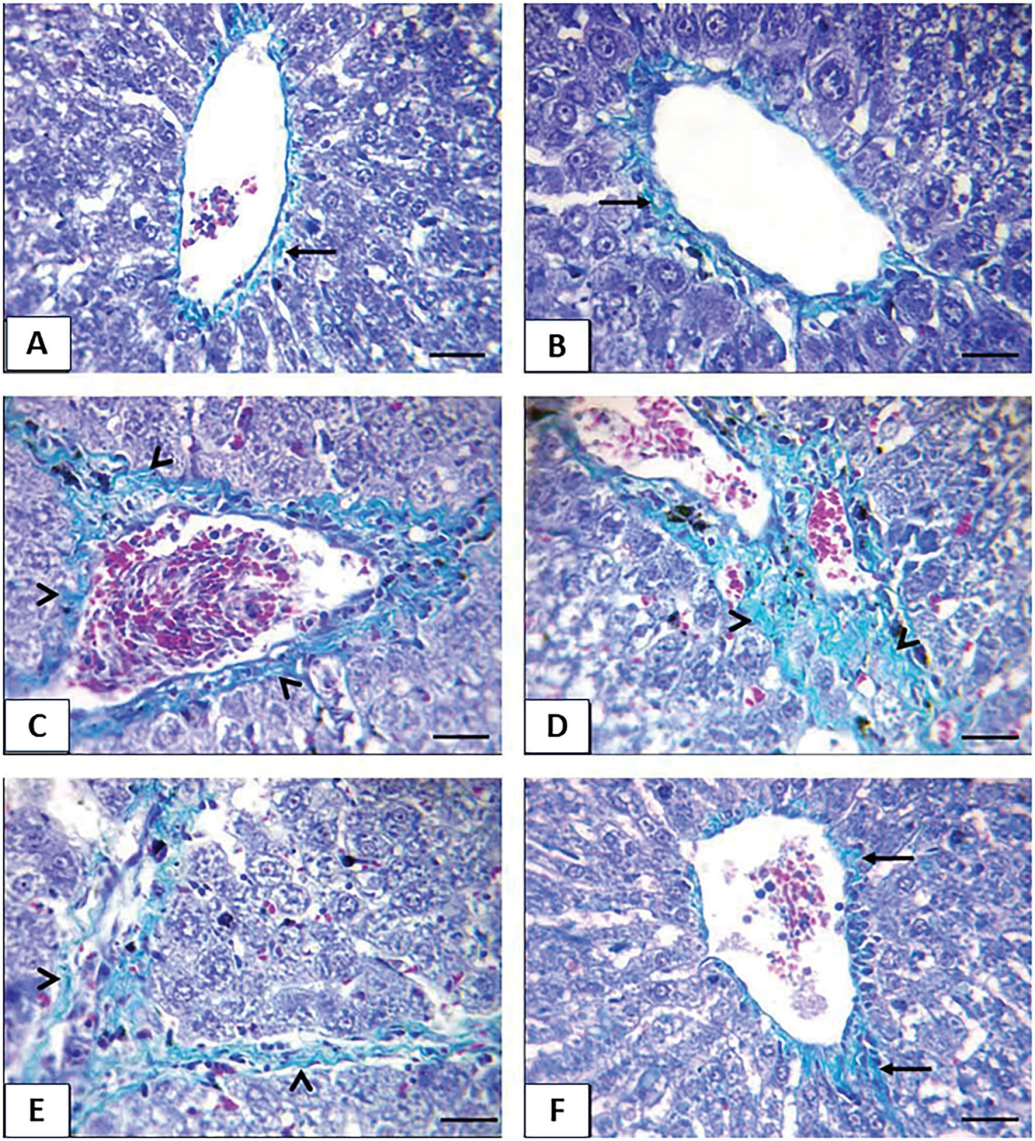
Photomicrograph of a rat liver section stained by Masson’s trichrome stain × 400. (**a**) Control rat (**b**) TQ treated rats (**c**, **d**, **e**) diabetic rats (**f**) TQ + diabetic treated rats showing fine light green collagen fibers (arrow), moderate and extensive thick collagen fibers (arrowheads). (Scale bar = 50 μm).

The obvious lesions of Masson’s trichrome in control and TQ-treated rats were fine light green collagen fibers (Fig. 9A and B). Diabetic rats displayed moderate and extensive thick collagen fibers around the central vein, in the portal area, and hepatic parenchyma (Fig. 9C, D, and E), while the Diabetic + TQ rats showed fine collagen fibers (Fig. 9F).

### Immunohistochemistry

**Figure 10:**
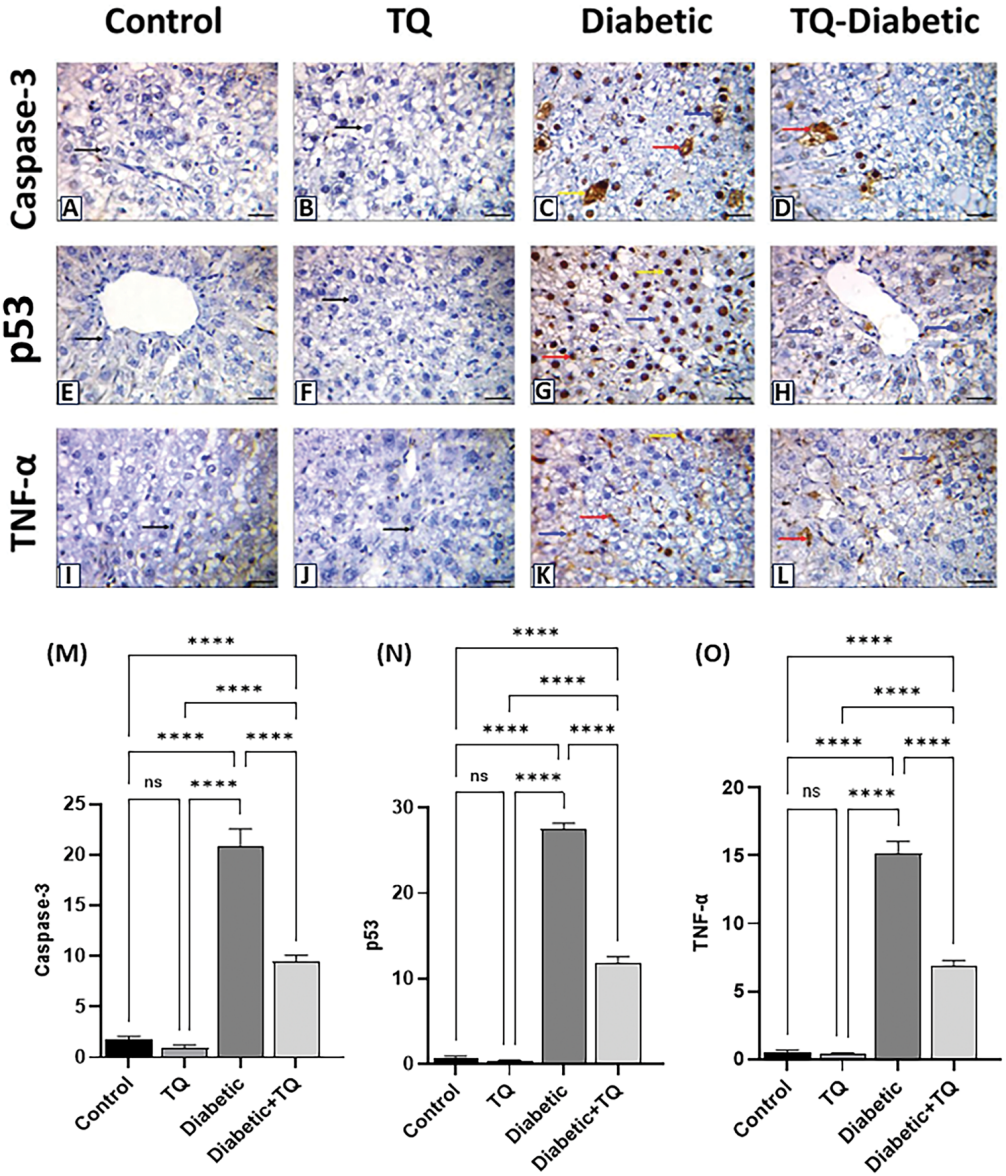
Photomicrographs of immunohistochemical staining × 400. Photomicrographs of immunohistochemical staining of caspase-3, p53, and tumor necrosis factor-alpha (TNF-*α*) proteins expression that show Negative immune-stained (black arrows), strong positive immune-stained (yellow arrows), moderate positive immune-stained (red arrows), weak positive immune-stained (blue arrows) cytoplasmic and nuclear hepatocytes (Scale bar = 50 μm). With Quantification of caspase-3, p53, and TNF-*α* expressios,, the immunohistochemical staining of caspase-3, p53, and TNF-*α* was measured as area percent (%) across 10 different fields/section, n = 8 rat/group. Data were analyzed with a one-way ANOVA followed by Tukey’s multiple comparison test. Data are expressed as the mean ± SD. ns = nonsignificant, ^*^P ˂ 0.05, ^**^P ˂ 0.01, ^***^P ˂ 0.001, and ^****^P ˂ 0.0001.

Nuclear and cytoplasmic immunostaining of hepatocytes of the non-diabetic control and TQ-administrated groups displayed a negative staining for the expression of caspase-3 protein (Fig. 10A and B). Conversely, diabetic animals exhibited moderate to strong positive brown immune reactions for caspase-3 protein (Fig. 10C). At the same time, Diabetic + TQ rats were weak immune brown staining for caspase-3 protein (Fig. 10D).

The immunohistochemical estimation of p53 protein of the hepatocytes of the control and TQ-treated animals displayed negative brown staining (Fig. 10E and F). On the contrary, diabetic rats exhibited strong to moderate nuclear-positive brown immunoreactivity of p53 protein expression (Fig. 10G). Nonetheless, there was weak nuclear immune brown staining in Diabetic + TQ rats (Fig. 10H).

The hepatic tissue immunostaining of the inflammatory TNF-*α* protein in control and TQ-administrated animals displayed a negative brown staining (Fig. 10I and J). In contrast, the diabetic group revealed moderate to strong positive brown reactions for TNF-*α* protein (Fig. 10K). In the Diabetic + TQ rats, there was less dense and weak brown staining (Fig. 10L).

The non-diabetic control and TQ-administrated groups displayed no significant alterations in the immune-stained area % of caspase-3 (Fig. 10M), p53 (Fig. 10N) and TNF-* α* (Fig. 10O). Compared with the normal control values, the diabetic group displayed a substantial (*P* < 0.001) enhancement in the immune-stained area % of caspase-3 (Fig. 10M), p53 (Fig. 10N) and TNF-*α* (Fig. 10O). The Diabetic + TQ-treated rats’ hepatic tissues displayed a marked reduction in immune-stained area % of caspase-3, p53 and TNF-* α* relative to the diabetic animals (Figs. 10M, N and O).

### Molecular docking assessment

**Figure 11:**
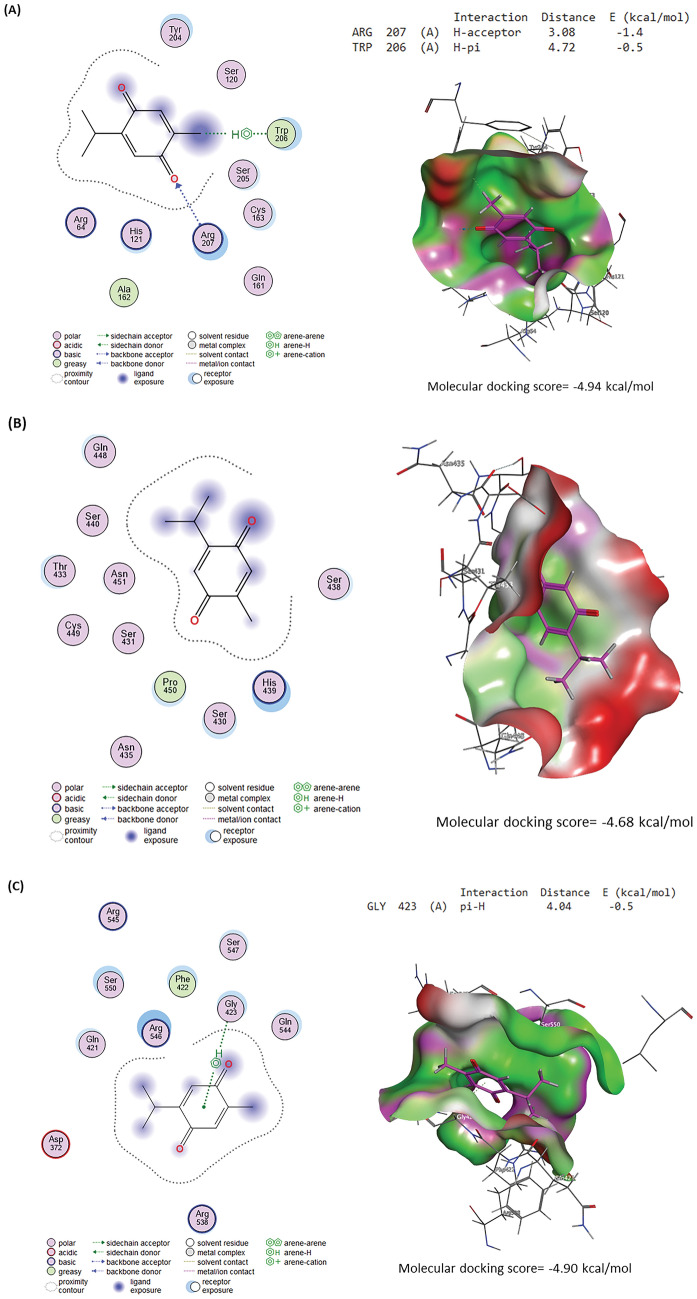

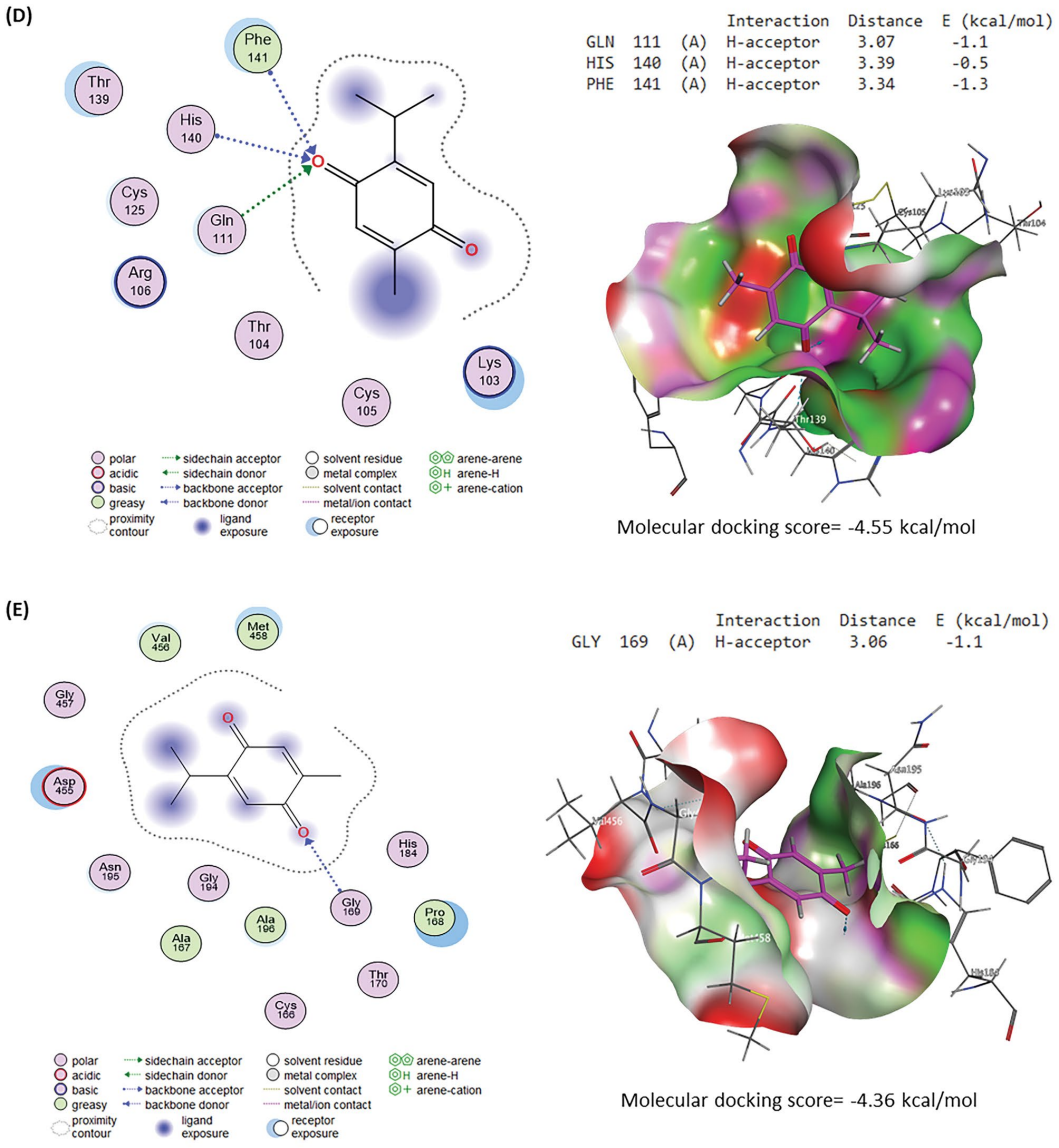
Molecular docking interaction of thymoquinone (TQ) and rats’ target proteins. **(A)** Caspase-3. **(B)** Interleukin-6 receptor (IL-6R). **(C)** Interleukin-1 receptor type 1 (IL1R1)**. (D)** Tumor necrosis factor receptor superfamily member 1A (TNFRSF1A). **(E)** Tumor necrosis factor receptor superfamily member 1B (TNFRSF1B).

Molecular docking interactions and scores of TQ against caspase-3, IL-6R, IL1R1, TNFRSF1A, and TNFRSF1B binding sites are illustrated in Fig. 11. TQ interacted with the binding site of caspase-3 with a binding energy of -4.94 kcal/mol by H-acceptor with ARG207 and H-pi with TRP206 residues (Fig. 11A). Figure 11B explores the interaction of TQ with the IL-6R binding site by -4.68 kcal/mol, binding energy. Furthermore, TQ interacted with GLY423 (pi-H) residue in the IL1R1 binding site by binding energy of -4.90 kcal/mol (Fig. 11C). By three H-acceptor bonds (GLN111, HIS140, and PHE141), TQ interacted with TNFRSF1A’s binding site by binding energy of -4.55 kcal/mol (Fig. 11D). Although TQ interacted with TNFRSF1B’s binding site by binding energy of -4.36 kcal/mol with GLY169 (H-acceptor) residue.

## Discussion

In many metabolic dysfunctions, antioxidant use is seen as an approach to reestablishing normal physiological balance^19^. DM is a systemic, metabolic, and endocrine illness defined mostly by hyperglycemia. It is also linked to glucose, lipid, and protein metabolism changes. This is because antioxidant processes within the body are being reduced and impaired^22,25^. Thymoquinone (TQ), the primary ingredient of *N. sativa*, is thought to be a hypoglycemic and anti-oxidative substance that might offset the side effects and enhanced cost of pharmaceutical medications^3,41^. The liver is the tissue utmost vulnerable to the effects of hyperglycemia-induced oxidative stress. Different processes, such as inflammation and oxidative stress, promote hepatocyte damage^20^. STZ is a diabetogenic and hepatotoxic substance that destroys the cell membrane of pancreatic beta-cells, breaks DNA, and interacts with many enzymes, counting glucokinase, causing insulin levels to drop dramatically^23^. It is critical to investigate the impact of anti-diabetic drugs in STZ-triggered hyperglycemia in research models. TQ was administered to animals in this experiment to evaluate their anti-diabetic and hepato-protective capabilities.

The current study displayed that STZ considerably reduced body weights in the third and fourth weeks of the experiment. The results are congruent with those of Almatroodi et al^42^. and Abdelrazek et al^22^. This might be due to decreased glucose and amino acid availability to cells of the body, which results in a deficiency of substrates required for the biosynthesis of the cells and can disrupt connected cellular metabolism, resulting in muscular wastage^2^. TQ treatment for diabetic rats caused a marked enhancement in the weight of the body relative to the diabetic animals. Present findings follow those of Abdelrazek et al^22^. and Abdel-Moneim et al^43^. This could support TQ's anabolic/antidiabetic action on diabetes-induced muscle atrophy due to glucose inaccessibility^16,42^.

Diabetic rats had lower blood insulin and HOMA *β*-cell function indicators than control rats. In contrast, the diabetes group had higher plasma glucose levels, HbA1c%, and glucose/insulin ratio than the control group. These data are consistent with those of Hafez et al^20^ and Faisal Lutfi et al^8^. HbA1c% is a widely used biomarker for assessing the severity of diabetes and its consequences^23^. This impact is caused by STZ's alkylating toxic activity on pancreatic islet *β*-cells, which limits insulin secretion, resulting in hyperglycemia^19^. TQ caused a significant drop and increase in plasma glucose and insulin levels compared to diabetic control rats. These findings agreed with those of Hofni et al^2^. and Abdelrazek et al^22^. TQ's plasma glucose lowering impact may demonstrate its insulinotropic activity, where it might cause partial regeneration of pancreatic islet β-cells, resulting in increased insulin synthesis and peripheral use^16^. TQ, in addition to its capacity to reduce intestinal glucose absorption, has an inhibiting impact on the expression of gluconeogenic enzymes and glucose synthesis in the liver^16,22^. Furthermore, it has the ability to stimulate adenosine monophosphate-activated protein kinase in the liver and muscles, blocking gluconeogenesis^42^.

TQ lowered blood glucose during the OGTT, which might be attributed to TQ's capacity to stimulate pancreatic *β*-cells insulin production^16^. Another factor is quinine's hypoglycemic feature, part of the TQ molecule's structure^41^. The mechanisms of action might be via inhibiting gluconeogenesis in hepatic tissue or glucose uptake into adipose tissues and muscles or by increasing glucose consumption in tissues^42^. As a result, unlike insulin and other synthetic medicines, TQ may be presumed to have anti-hyperglycemic properties^22,43^ without causing any undesirable hypoglycemia.

STZ promotes experimental hyperglycemia in rats, increasing ALT, AST, ALP, CYP450, and hepatic G6Pase activity, which is a strong sign of liver injury. AST and ALT are closely related to converting amino acids to keto acids and are known to rise with DM^43^. CYP450 isoforms are a class of enzymes that can catalyze various enzymatic processes, including xenobiotic metabolism^17,44^. G6Pase is a critical and rate-limiting enzyme in gluconeogenesis, as demonstrated by an experiment in which G6Pase knockout mice had symptoms such as hyperlipidemia and lactic acid buildup^45^. The elevated activities of serum markers of liver function (ALP, ALT, AST, CYP450, and G6Pase) in the current investigation suggested that hepatic damage could be caused by the generation of hyperglycemia by infusing STZ. The rise in AST and ALT activity could be attributed to impairment of the cells in the hepatic tissue due to STZ-induced diabetes^20^. The present experiment revealed that treating diabetic rats with TQ reduces liver function enzyme activity to virtually normal levels, indicating that TQ protects liver cellular functioning. Previous research has shown that TQ positively affects hepatic key enzymes in diabetic rats^3,41^. It was also previously revealed that insulin has a role in downregulating CYP450 and G6Pase^22^, which could explain why TQ therapy enhanced insulin levels in diabetic rats.

According to the present experiment, within the diabetic control group, STZ caused dyslipidemia (lower HDL-C and higher TC, LDL-C, TAG, and AIP). These results agreed with those of Abdelrazek et al^22^. DM-induced dyslipidemia develops as a result of hyperglycemia and insulin deficiency, which enhances lipolysis and fatty acid production from adipose tissue to the circulation with changes in the metabolism, raising LDL-C, TC, and TAG values and predisposing to cardiovascular dysfunction^43^. TQ administration improves the diabetes-induced aberrant lipid profile, which is in line with the results of Abdelrazek et al^22^. and Abdel-Moneim et al^43^. TQ's beneficial effects on DM-induced dyslipidemia might be attributed to its stimulation of the activity of hepatic arylesterase, antioxidant properties, and adjusting impacts on the genes influencing cholesterol metabolism^1,3^.

Lipid peroxidation is a symptom of cellular impairment caused by ROS. In addition to MDA generation, excessive concentrations of ROS promote lipid peroxidation of the cellular membrane^2,46^. The true procedure of STZ-administrated DM in animals implicates ROS formation, DNA alkylation, and an increase in NO production in *β*-cells of the pancreas. Decrease of *β*-cell mass and function is the primary cause of diabetes and initiates dysfunction of the pancreatic *β*-cell^22^. Diabetes-related oxidative/nitrosative stress causes glucose autoxidation, lipid peroxidation, protein glycation, and decreased activity of antioxidant enzymes^1^. Lipid peroxidation might be produced by oxidative stress with enhanced mitochondrial uncoupling protein expression, activating cytokines, ligands, and growth factors, resulting in fibrosis and inflammation^47^. MDA and nitrite levels in the hepatic tissue were considerably greater in STZ-administrated diabetic animals. TQ administration also markedly reduced hepatic oxidative/nitrosative stress indicators, suggesting that TQ possess an important influence as a hepatic protective agent in contradiction to STZ diabetic mice, principally through its antioxidant characteristics^2,42^.

The present experiment considered the effect of TQ on oxidative stress of the liver in STZ DM in male rats. Fundamental processes of diabetes-induced free radicals and oxidative stress have been widely studied^19,20^. Diabetic rats had considerably lower hepatic GSH levels, activities of T-SOD, and CAT because of hyperglycemia and hyperlipidemia, which reduce the natural antioxidant activities and stimulate the generation of free radicals^3,8^. TQ treatment increased the antioxidant reserve of GSH levels as well as T-SOD and CAT activities in hepatic tissues, supporting the findings of Almatroodi et al^42^., Abdelrazek et al^22^., and Abdel-Moneim et al^26^. The inclusion of quinine in the TQ structure confirms its antioxidant capabilities. Quinine improves the admission to cellular and subcellular components, facilitating ROS removal^16^. TQ reduced oxidative stress by inhibiting nonenzymatic lipid peroxidation^2,41^. Furthermore, TQ's hypoglycemic impact enhances its antioxidant effect by regulating hyperglycemia-induced ROS.

These findings were confirmed in our work by immunostaining for the hepatic tissue inflammatory TNF-α protein of diabetic rats, which demonstrated a moderate to strong positive brown reaction for TNF-α protein with a marked increase in the immunohistochemical staining of TNF-α. Inflammation plays a significant role in the progression of diabetes and is consequently related to increased resistance to insulin and decreased responsiveness in the target tissues of insulin^18^. Furthermore, pro-inflammatory cytokines (IL-6, TNF-*α*, and IL-1*β*) and inflammatory markers (CRP) are important markers of pancreatic *β*-cell dysfunction and insulin resistance, all of which provide the DM progression^3,42^. These findings were confirmed in our work by immunostaining for the inflammatory protein of TNF-*α* in the liver of diabetic rats, which demonstrated a moderate to strong positive brown reaction for TNF-α protein. Circulating values of most proinflammatory cytokines are often raised in DM, owing to hyperglycemia, which raises cytokine levels in the blood via an oxidative mechanism^48^. It was discovered that diabetic liver had considerably enhanced pro-inflammatory cytokines (TNF-*α,* IL-1*β*, and IL-6) protein production and the inflammatory infiltration of macrophages^1^. TNF-*α* was significantly elevated in the STZ diabetic group, with enhancing DNA destruction in the hepatic tissue, which also substantially enhanced the stimulation of nuclear factor kappa B, Janus kinases-signal transducer and stimulator of transcription, and c-Jun N-terminal kinases signaling pathways^16^. TQ treatment reduced liver TNF-*α* protein levels and immunostaining significantly. According to our findings, STZ may trigger an inflammatory mechanism that contributes to diabetic liver damage^20^. Previous research has shown that TQ decreases pro-inflammatory markers (IL-6, TNF-*α*, and cyclooxygenase-2)^18,49^. Because of its anti-inflammatory capabilities, TQ suppressed the overproduction of pro-inflammatory markers (IL-6, IL-1*β*, and TNF-*α*), an inflammatory indicator (CRP), and immunostaining of TNF-*α* in STZ-administrated diabetic rats^2,41^. These findings were also validated by the current study's molecular docking findings. TQ displayed affinity to interact with and block the binding sites of IL-6R, IL1R1, TNFRSF1A, and TNFRSF1B, which may explain TQ's anti-inflammatory effects, as recognized by Alyami and Al-Hariri^16^.

Microscopic examination of normal control and TQ hepatic tissue revealed normal histological structure, with undamaged hepatic architecture in hepatic lobules, portal regions, and hepatic vasculature. STZ-induced diabetic rats had diffuse hepatic vasculature congestion, diffuse hepatic hydropic degeneration, and intense mononuclear cell infiltrations in the portal area, which was accompanied by a moderate or extensive thick collagen fiber around the portal vein and hepatic parenchyma^50^. STZ has a degenerative effect on hepatocytes, in addition to free radical buildup and damage due to oxidative stress^45^. The pathophysiology of diabetic hepatic injury may be due to inadequate insulin production or insulin resistance caused by DM, which causes hyperglycemia and hyperlipidemia due to carbohydrate metabolism disruption^47^. Diabetic rats given TQ had essentially normal histoarchitecture. Because of its antioxidant, anti-inflammatory, and hypoglycemic properties, TQ is a promising candidate for managing diabetes complications^16^.

Apoptosis, or programmed cell death, is a physiological mechanism of the body that performs a critical function in maintaining the body's environmental stability. It is a biomarker that responds to persistent stress. Excessive or insufficient apoptosis would be harmful to the body. It is also considered an important defensive mechanism because it regulates many effector cells^20^. A pro-apoptotic marker (caspase-3) is an effector protein in the intrinsic and extrinsic apoptotic mechanisms^47^. The immunohistochemical and quantitative analyses of our experiment revealed that the STZ-diabetic group had considerably higher caspase-3 immunopositive hepatic cell counts. Faisal Lutfi et al^8^. discovered that STZ-induced oxidative damage in male diabetic rats' hepatic tissues indicated a high presence of caspase-3 positive cells and a low expression of Bcl2. TQ reduced the expression of caspase-3 and p53 proteins in the hepatic tissue of diabetic rats, implying that TQ protects the liver by inhibiting apoptosis and inflammation^41^; this was also confirmed by the findings of the current study's molecular docking, which revealed that TQ interacts with and inhibits the caspase-3 binding site.

ROS causes DNA damage and stimulates a response to it, leading to the overexpression of p53. Chronic hyperglycemia followed by an increase in cytoplasmic glucose concentration causes C-terminal glycosylation of the tumor suppressor p53, which then activates the transcriptional activator p53, resulting in its translocation to the nucleus and the initiation of transcription of several p53-dependent genes^21,51^. The overall response of many cell types to DNA-damaging chemicals is typically characterized by p53 protein overexpression^52^. The tumor suppressor gene p53 governs cell cycle progression, induces apoptosis, and is integrated in progression of diabetic complications in response to DNA damage at the G1/S checkpoint^51^. Diabetes patients had higher levels of p53 in their livers, which was associated with the resistance to insulin^21^. Because TQ modulates blood glucose concentrations in diabetic animals, TQ treatment lowered immunostaining of p53, which could indicate that TQ has an anti-apoptotic effect in the handling of DM^2,42^.

## Conclusion

To conclude, TQ has anti-diabetic, anti-inflammatory, anti-apoptotic and hepatic protective actions via modulating blood glucose and lipid profile, as well as enhancing liver functioning, hepatic histoarchitecture and oxidative stress status. In the current work, we found that TQ displayed abilities to interact and inhibit the binding site of caspase-3, interleukin-6 receptor, interleukin-1 receptor type 1 and TNF receptor in rats following the molecular docking modeling. Overall, TQ alleviates most of the alterations seen in diabetic animals and effectively ameliorates the harm caused by STZ; consequently, it’s recommended to be integrated with the diabetes mellites management protocols. TQ's detailed molecular mechanism in diabetes management must be further investigated.

## Data Availability

All data generated or analyzed during this study are included in this published article.

## Abbreviations

AIP: Atherogenic index
ALP: Alkaline phosphatase
ALT: Alanine aminotransferase
ANOVA: Analysis of variance
AST: Aspartate aminotransferase
CAT: Catalase
CRP: C-reactive protein
CYP450: Cytochrome p450
DM: Diabetes mellitus
G6Pase: Glucose-6-phosphatase
GSH: Reduced glutathione
HbA1c: Glycosylated hemoglobin
HDL: High density lipoprotein
HOMA-*β*: Homeostasis model assessment of β-cell function
i.p.: Intraperitoneal
IL-1*β*: Interleukin-1*β*
IL-6: Interleukin-6
LDL: Low density lipoproteins
MDA: Malondialdehyde
NO: Nitric oxide
OGTT: Oral glucose tolerance test
ROS: Reactive oxygen species
SD: Standard deviation
STZ: Streptozotocin
TAG: Triglycerides
TC: Total cholesterol
TNF-*α*: Tumor necrosis factor-alpha
T-SOD: Total superoxide dismutase
